# Exploring Impact of Marijuana (Cannabis) Abuse on Adults Using Machine Learning

**DOI:** 10.3390/ijerph181910357

**Published:** 2021-10-01

**Authors:** Jeeyae Choi, Joohyun Chung, Jeungok Choi

**Affiliations:** 1 School of Nursing, University of North Carolina, Wilmington, NC 28403, USA; 2 College of Nursing, University of Massachusetts, Amherst, MA 01002, USA; joohyunchung@umass.edu (J.C.); jeungokc@nursing.umass.edu (J.C.)

**Keywords:** marijuana abuse, machine learning, substance abuse, health impact assessment

## Abstract

Marijuana is the most common illicit substance globally. The rate of marijuana use is increasing in young adults in the US. The current environment of legalizing marijuana use is further contributing to an increase of users. The purpose of this study was to explore the characteristics of adults who abuse marijuana (20–49 years old) and analyze behavior and social relation variables related to depression and suicide risk using machine-learning algorithms. A total of 698 participants were identified from the 2019 National Survey on Drug Use and Health survey as marijuana dependent in the previous year. Principal Component Analysis and Chi-square were used to select features (variables) and mean imputation method was applied for missing data. Logistic regression, Random Forest, and K-Nearest Neighbor machine-learning algorithms were used to build depression and suicide risk prediction models. The results showed unique characteristics of the group and well-performing prediction models with influential risk variables. Identified risk variables were aligned with previous studies and suggested the development of marijuana abuse prevention programs targeting 20–29 year olds with a regular depression and suicide screening. Further study is suggested for identifying specific barriers to receiving timely treatment for depression and suicide risk.

## 1. Introduction

Marijuana is the most commonly used illegal substance worldwide [1,2,3,4,5]. In recent decades, the rate of marijuana users has increased in young adults and pregnant women in the US [6]. The Substance Abuse Center for Behavioral Health Statistics and Quality reported that more than 11.8 million young adults used marijuana in 2018 [7]. Alarming signs of marijuana use among high schoolers were reported in 2019. Twenty-eight percent of second-year high school students indicated that they had used marijuana in the previous year and 35% of senior students indicated they used it during the year prior to 2019 [8]. The current climate of legalizing and decriminalizing marijuana use would contribute to further increments [3,9,10].

Marijuana use could lead to addiction, although it is often questioned and treated as not a serious addiction problem [11]. However, marijuana is highly addictive with serious adverse effects [3,12,13,14]. Studies indicate marijuana use is associated with various psychosocial and medical problems in young adults [10,15,16]. Berenson explained the association between marijuana, mental illness, and violence [17]. Zehra and colleagues’ study showed repeated marijuana use could cause neurobiological changes in the brain, resulting in addictive behavior and withdrawal symptoms [18]. A study confirmed that cognitive function impairment is caused by marijuana addiction [19]. In addition, marijuana use lowers birth weight and has destructive impacts on fetal growth in pregnant women [20]. The American College of Obstetricians and Gynecologists (ACOG) announced that a nursing mother’s marijuana use could affect a baby’s brain development because it can be accumulated in breast milk to high concentrations. ACOG is discouraging nursing mothers from using marijuana [21].

Studies indicated that cannabis (marijuana) use is strongly associated with depression and anxiety disorders [22,23,24]. A study of medical marijuana showed that the use of cannabis during adolescence is related to depression and suicidality in adult life and regular use of cannabis at a young age increases the risk of depression [25]. Suicidal behavior is affected by biological, psychiatric, and psychosocial determinants and substance use disorders. Suicidal ideation and attempts are associated with illicit substances such as cocaine, marijuana, and methamphetamine [26]. Research has also shown that marijuana aggregates mental health and influences repeated attempts of suicide in high schoolers [27,28].

Cannabidiol (CBD) and tetrahydrocannabinol (THC) are two fundamental chemicals in marijuana. CBD is shown to reduce anxiety, depression, and seizures, while THC is known to produce high sensation and intoxicating effects [8,29]. Medical marijuana (cannabis) is extracted from plants of the genus cannabis to treat specific diseases and symptoms using CBD’s therapeutic effects [30,31]. For example, it shows improvement in seizures, Dravet syndrome, Lennox-Gastaut syndrome, pain, spasticity, inflammatory bowel disease, nausea, anorexia, depression, and anxiety [29,30,32]. However, researchers and clinicians remain concerned about the risks caused by medical marijuana and insist upon assessment of its impact on adults [18,33]. Bridgeman and Abazia acknowledged the beneficial effects of CBD in medical marijuana yet simultaneously emphasized how their efficacy in alleviating symptoms or disease was not well established [30]. There are increased concerns about the amount of THC that causes intoxication and addiction in medical marijuana due to no regulation [34,35]. Pure CBD has been proven to be safe in a research environment, which may not be possible to replicate in commercial CBD products [32]. Cascardo reported that many clinicians are not comfortable with their supervising role when there is limited clinical research available [14].

Marijuana is the most commonly used substance after alcohol and tobacco in the US [8]. Recent legalization of marijuana uses for medical or recreational purpose decreases the perception of risks of marijuana, expecting an increase of marijuana users. Currently, there is a lack of knowledge on how marijuana is affecting psychosocial and medical conditions of adults. The purpose of this study was to explore and analyze characteristics of adults who abuse marijuana (20–49 years old) on behaviors and social relations impacting depression and suicide risk using machine-learning algorithms.

## 2. Materials and Methods

### 2.1. Research Design

An exploratory machine-learning approach was adopted and applied to the National Survey on Drug Use and Health data sets. Specific machine-learning algorithms used in the study were logistic regression, Random Forest, and K- Nearest Neighbor (K-NN).

### 2.2. Data Source

We used the publicly available 2019 National Survey on Drug Use and Health (NSDUH) data sets collected electronically. The primary purpose of this survey was to measure the prevalence and find correlations between substance (illicit drugs, alcohol, and tobacco) use and mental health issues in the US. This was the 39th in a series and participants were citizens of the U.S., including those living on military bases, who were 12 years of age or older at the time of the survey.

The Center for Behavioral Health Statistics and Quality (CBHSQ) within the Substance Abuse and Mental Health Services Administration (SAMHSA) conducted the 2019 NSDUH. Data were collected from 50 states and the District of Columbia using an audio computer-assisted self-interviewing method. The data are publicly available and had already undergone a confidentiality review. The full analytic file of all participants was treated using a statistical disclosure limitation method called MASSC, which consists of the following four major steps: (1) Micro Agglomeration, (2) optimal probabilistic Substitution, (3) optimal probabilistic Subsampling, and (4) optimal sampling weight Calibration. Personal Identifiable Information (PII) such as name, phone number, and address on the file was removed. A total of 56,136 participants completed the NSDUH in 2019, which was reviewed and approved by RTI’s Institutional Review Board under federal regulation [36,37].

### 2.3. Data Collection

We selected participants who were 20 to 49 years old and classified as having marijuana dependency based on participants’ responses related to marijuana uses and behaviors. We applied the mean imputation for missing data and analyzed them using SAS v9.4 and SAS Enterprise Miner v15.1 (SAS Institute Inc., Cary, NC, USA). The missing data rate was 21.0% based on missing data (n = 48,957) and expected data (n = 233,132).

**Figure 1:**
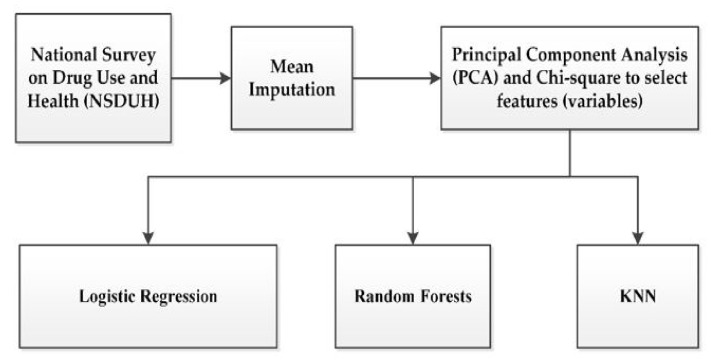
Overall process of building predictive models.

To explore detailed characteristics of adult marijuana users’ behavior and social relation, we focused on depression and suicide risk, mental health conditions. We identified and measured the effects of important variables using Principal Component Analysis and Chi-square. Depression and suicide risk prediction models were built using three machine-learning algorithms (logistic regression, Random Forest, and K-NN) and their performance was measured. The data were split into two parts for training (70%) and testing (30%) in the ratio of 70:30 for evaluating models’ performances. Figure 1 shows the process of building the prediction models.

### 2.4. Data Preprocessing and Defining Labels

#### 2.4.1. Feature (Variable) Selection

We removed irrelevant variables such as respondent identification and recoded variables. In a total of 2741 variables, 851 irrelevant variables were removed, and 1890 variables remained. Principal Component Analysis (PCA) and Chi-square [38] were used to select features (variables). PCA is one of the popular tools for feature selection and reduction of dimensionality in machine learning [39,40]. It removes correlated features, improves algorithm performance, and reduces overfitting [41].

#### 2.4.2. Imputation for Missing Data

We used the mean imputation method for missing data because it is straightforward and preserves the mean of the observed data when the data are missing at random [42,43]. A total of 334 variables were reviewed, and 93 variables were included in the final model.

#### 2.4.3. Labeling of Risk for Depression

The NSDUH assessed major depressive episodes with six questions, which were: (1) How often did you feel nervous in the past 30 days? (2) How often did you feel hopeless in the past 30 days? (3) How often did you feel restless in the past 30 days? (4) How often did you feel sad and that nothing could cheer you up in the past 30 days? (5) How often did you feel that everything was an effort in the past 30 days? and (6) How often did you feel down on yourself, no good or worthless in the past 30 days? When a participant responded as ‘all the time’ to any of these six variables, it was considered as they had depression in this study.

#### 2.4.4. Labeling of Suicide Risk

Three suicide-related variables were used to label. They were: (1) Thought of suicide at any time in the past 12 months, that is from [the date 12 months prior] up to and including today, did you seriously think about trying to kill yourself? (2) Plan of suicide, during the past 12 months, did you make any plans to kill yourself? and (3) Attempt to suicide, during the past 12 months, did you try to kill yourself? Any participants indicating ‘yes’ to these questions were considered as risk for suicide in this study.

### 2.5. Machine Learning Algorithms

#### 2.5.1. Logistic Regression

Logistic regression is the most widely used machine-learning algorithm for binary outcomes [44]. It is based on logistic function, one type of sigmoid function, which converts real-valued continuous inputs into categorical values. This algorithm assumes a linear relationship between the logarithm of the odds of the outcome and the predictors as equivalent [45].

#### 2.5.2. Random Forest (RF)

Random Forest is an ensemble machine-learning algorithm that has a computational efficiency over larger data sets. This algorithm randomly selects a subset of variables and constructs many decision trees. Every individual tree splits its nodes to get a class prediction. Strengths of RF are low bias, high variance, and low correlation between constructed trees [46,47].

#### 2.5.3. K-Nearest Neighbor (KNN)

KNN is a simple machine-learning algorithm that finds the closest data from a query data point depending on k value. Data are classified by the distance to others. It is a non-parametric algorithm and its calculation time is short due to no training period being needed [48,49].

### 2.6. Measurement of Prediction Model Performances

Sensitivity, Specificity, Accuracy, AUC (area under the curve), Precision, and F1 score were used to measure the performances of prediction models and were calculated based on confusion matrix. Sensitivity is referred to as the true positive rate. From a confusion matrix, the four terms were defined as (1) True positive (TP) = the number of cases correctly identified as the presence of the outcome, (2) False positive (FP) = the number of cases incorrectly identified as the presence of the outcome, (3) True negative (TN) = the number of cases correctly identified as the non-presence of the outcome, (4) False negative (FN) = the number of cases incorrectly identified as the non-presence of the outcome.

Sensitivity = TP/(TP + FN)(1)

Specificity is the true negative rate (TNR), that is, the proportion of the actual cases that are correctly predicted as negative.

Specificity = TN/(TN + FP)

Precision is the ratio of correctly predicted positive observations to the total predicted positive observations.

Precision = TP/TP + FP

Accuracy is the ratio of correctly classified observations to the total number of observations.

Accuracy = (TP + TN)/(TP + TN + FP + FN)

F1 score is a measure of the weighted average of precision and recall.

F1 score = 2 × (recall × precision)/(recall + precision)

The Area Under the Curve (AUC) is the measure of the ability of a classifier to distinguish between classes and is used as a summary of the receiver operating characteristic (ROC) curve.

## 3. Results

A total of 698 participants were identified as marijuana dependent in the past year. The majority were between 20–29 years old (n = 548, 78.51%), had never been married (n = 573, 82.09%), employed (n = 487, 82.96%), and covered by any health insurance (n = 560, 81.04%). More than half were male (n = 421, 60.32%), half were NonHispanic white (n = 364, 52.15%), about one-third had some college credit but no degree (n = 249, 35.67%), and about one-quarter of them reported that their family income was more than $75,000 (n = 175, 25.07%). Table 1 shows characteristics of participants in this study.

### 3.1. Features (Variables) Identified

**Figure 2:**
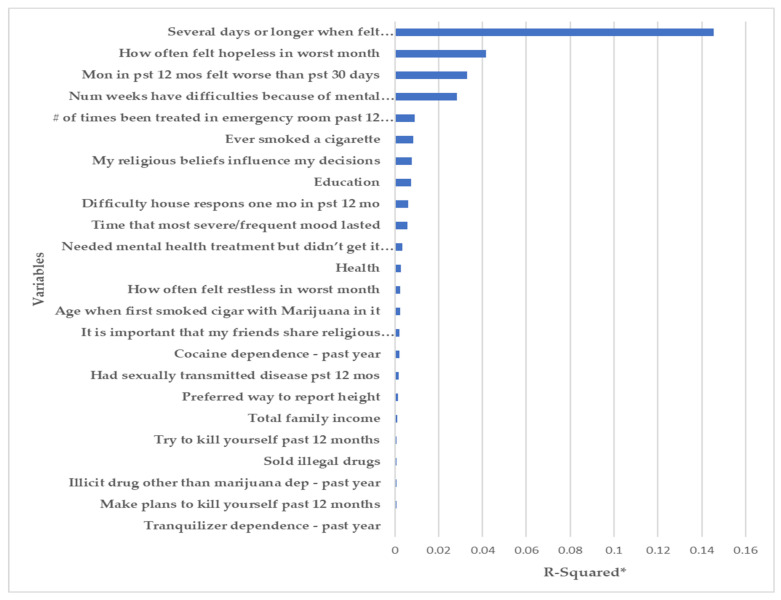
Importance plot for depression (* a larger R-squared value means that a variable explains a larger percentage of the variation in the outcome variable).

**Figure 3:**
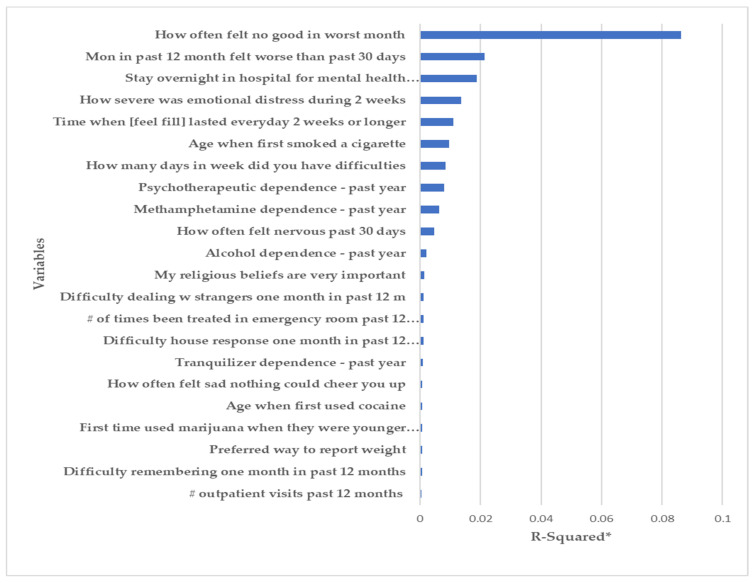
Importance plot for the suicide risk (* a larger R-squared value means that a variable explains a larger percentage of the variation in the outcome variable).

The relative importance plots of the input variables were ranked by the Chi-square criteria and are depicted in Figure 2 and Figure 3.

#### 3.1.1. Depression

A total of 271 out of 698 participants (38.8%) indicated ‘all the time’ to risk for depression questions. The relative importance plot (Figure 2) shows that “Several days or longer when felt sad/empty/depressed” was the highest ranked variable for depression risk prediction models. “How often felt hopeless in the worst month” and “Months in past 12 months felt worse than past 30 days” were ranked as the next highest variables.

#### 3.1.2. Suicide Risk

A total of 180 out of 698 participants (25.8%) reported ‘yes’ to questions related to suicide risk. The relative importance plot (Figure 3) shows that “How often felt hopeless in worst month” was the highest ranked variable for suicide risk prediction models. “Months in past 12 months felt worse than past 30 days” and “Stay overnight in hospital for mental health treatment past 12 months” were ranked as the next highest variables.

### 3.2. Measurement of Prediction Model Performances

Sensitivity, Specificity, Accuracy, AUC (area under the curve), Precision, and F1 score were calculated. The comparative results of three algorithms (logistic Regression, RF, KNN) with 89 attributes are summarized in Table 2 and Table 3.

#### 3.2.1. Depression

Table 2 is a summary of performance of prediction. RF shows the highest accuracy of 0.773, while the logistic regression shows the lowest accuracy of 0.635. Although KNN and RF show similar performance, RF shows higher accuracy than KNN. KNN shows higher precision than RF. Logistic regression shows relatively poor performance in depression risk prediction.

#### 3.2.2. Suicide Risk

Table 3 is a summary of performance of prediction. RF shows a great performance in accuracy (0.998) and AUC (1.0). In suicide risk prediction, logistic regression and KNN models show the similar performance.

#### 3.2.3. Summary of the ROC Curves

**Figure 4:**
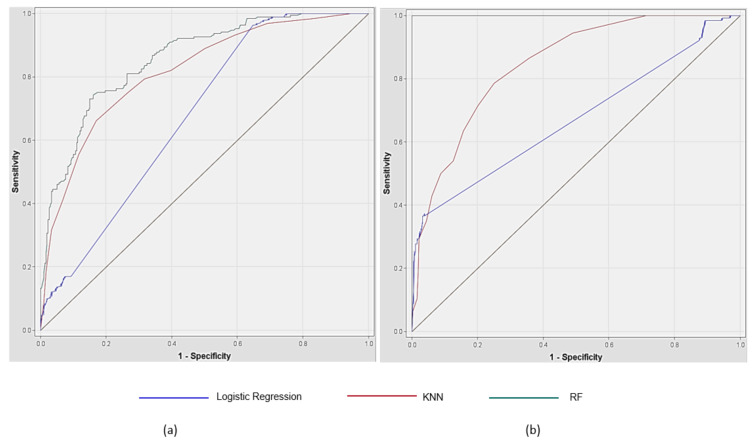
ROC Curves of depression and suicidal risk prediction; (**a**) Depression; (**b**) Suicide.

The ROC curves in Figure 4 were plotted for depression and suicidal risk prediction. RF shows great performance in both depression and suicidal risk prediction, while logistic regression shows fair performance in both depression and suicidal risk prediction.

## 4. Discussion

We identified demographic characteristics of adults (20–49 years old) who abuse marijuana. The identified demographic characteristics for most of them were 20–29 years old, never been married, employed, and covered by a health insurance, as aligned with other study findings [50,51]. Overall depression and suicide risk prediction models built by RF and KNN showed good performance (Accuracy = [0.740–0.998], AUC = [0.816–1.0]) but logistic regression showed poor performance. Among the three machine-learning algorithms, models built by RF showed excellent performance (Accuracy = [0.773–0.998], AUC = [0.857–1.0]).

### 4.1. Impact on Mental Condition

In 2019, the US Census Bureau estimated 328.2 million people in the U.S. and 129.61 million of 20–49 age group (39.5%) [52]. Analyzing the social relations and behaviors of this same group in this study is valuable. The risk variables identified in both prediction models were (1) months in past 12 months felt worse than past 30 days, (2) number of times been treated in the emergency room past 12 months, (3) difficulty in taking care of household responsibilities 1 month in past 12 months, and (4) tranquilizer dependence past year. These indicate that people had been under emotional distress for at least 12 months but had not received proper treatment. These findings are particularly notable since most were employed and covered by health insurance, suggesting finance and healthcare accessibility were not major barriers. The “religious belief” was identified as an influential social relation variable in both prediction models.

Marijuana use such as “smoking cigarette with marijuana in it” and “first time use of marijuana was younger than 20 years old” were identified as variables to influence depression or suicide risk, but not as high as expected. This implies that other factors than marijuana use affected their depression and suicide risk mental state more. Alcohol consumption is known to be associated with depression and suicide risk [27,50,53,54], although its affects as an influential variable to predict depression were beyond the scope of the current study.

Both prediction models showed cocaine and methamphetamine (meth) as a risk variable in depression and suicide, respectively. They are the same type of stimulant but have different effects on the human body. Cocaine is a plant-driven substance and meth is synthesized using various chemicals. Meth increases more dopamine than cocaine, resulting in stronger and longer lasting effects. This study results showed that cocaine was also linked with depression and both cocaine and meth were linked with suicide risk, which are aligned with previous studies [27,55,56,57].

### 4.2. Implication for Practice

This study supports earlier published evidence and will aid future investigators in applying a better-informed use of variables. Results revealed that most participants were employed and covered by health insurance; however, they still did not seek or receive proper care to prevent depression or suicide risk. In addition, “religious belief” was identified as a risk variable in both depression and suicide risk prediction models but its impact on this mental condition is not clear. Further study is strongly needed to find reasons and level of impact.

### 4.3. Limitations

National Survey on Drug Use and Health (NSDUH) data are based on self-reports of drug use and dependencies. Although NSDUH procedures were designed to strengthen participants’ honesty and recall, the degree of underreporting and overreporting of information was unknown. This survey is cross-sectional, measuring responses at a single snapshot in time, hence, overlooking the development of abuse and dependency behaviors as they develop over time. Although the excluded population was only 3%, their inclusion may generate different results. If other feature selections and prediction model algorithms were used, there may be different risk variables showing different performances.

## 5. Conclusions

We successfully identified unique characteristics of adults (20–49 years old) who abused marijuana, using publicly available data sets. Well-performing depression and suicide risk prediction models were built using three machine learning algorithms, logistics regression, RF, and KNN. The identified most influential risk variables in models could guide the focus of future marijuana abuse prevention studies. For example, development of marijuana abuse prevention programs targeting the ages of 20–29 group with a regular depression and suicide risk screening. Further study is needed to identify specific barriers of receiving timely treatments for depression and suicide risks when they are well financed and cover a health insurance and level of religious belief impact on depression and suicide risk. Results show that machine learning is a useful tool to explore the impact of marijuana abuse on adults (20–49 years old).

## Figures and Tables

**Table 1:** Characteristics of participants who abuse marijuana (n = 698).

Characteristics		n (n = 698)	%
Gender	Female	277	39.68
	Male	421	60.32
Age	20–29	548	78.51
	30–34	70	10.03
	35–49	80	11.46
Race	NonHispanic White	364	52.15
	NonHispanic Black or African American	100	14.33
	NonHispanic	19	2.72
	Native American/Alaska Native		
	NonHispanic	3	0.43
	Native HawaiianI/Other Pacific Islander		
	NonHispanic Asian	25	3.58
	NonHispanic more than one race	48	6.88
	Hispanic	139	19.91
Education	5th–12th grade completed, no diploma	59	8.45
	High school diploma/GED	204	29.23
	Some college credit, but no degree	249	35.67
	Associate degree	64	9.17
	College graduate or higher	122	17.48
Family income	Less than $10,000	90	12.89
	$10,000–$19,999	94	13.47
	$20,000–$29,999	71	10.17
	$30,000–$39,999	76	10.89
	$40,000–$49,999	88	12.61
	$50,000–$74,999	104	14.90
	$75,000 or more	175	25.07
Marital status	Married	83	11.89
	Widowed	2	0.29
	Divorced or Separated	40	5.73
	Never Been Married	573	82.09
Employment	Employed	487	69.77
	Unemployed	100	14.33
	No response	111	15.90
Health Insurance	Covered by any Health Insurance	560	80.23
	Not covered	131	18.77
	No response	7	1.00

**Table 2:** Performance of depression risk prediction.

Model	Sensitivity	Specificity	Accuracy	95% CI for Accuracy	AUC	Precision	F1 Score
Logistic Regression	0.690	0.632	0.635	0.593–0.678	0.675	0.106	0.184
RF	0.771	0.773	0.773	0.753–0.810	0.857	0.587	0.667
KNN	0.751	0.732	0.740	0.701–0.779	0.816	0.640	0.691

**Table 3:** Performance of suicide risk prediction.

Model	Sensitivity	Specificity	Accuracy	95% CI for Accuracy	AUC	Precision	F1 Score
Logistic Regression	0.771	0.815	0.810	0.775–0.845	0.674	0.373	0.503
RF	1.0	0.997	0.998	0.993–1.002	1.0	0.992	0.996
KNN	0.711	0.826	0.808	0.773–0.843	0.845	0.429	0.535

## Data Availability

Publicly available data sets were analyzed in this study. The data can be found here: https://www.samhsa.gov/data/release/2019-national-survey-drug-use-and-health-nsduh-releases (accessed on 15 March 2021).
